# A COSMIN Systematic Review of Transition Readiness Assessment Tools for Adolescents with Type 1 Diabetes

**DOI:** 10.3390/healthcare14050639

**Published:** 2026-03-03

**Authors:** Valentina Vanzi, Maddalena De Maria, Gabriele Caggianelli, Dhurata Ivziku, Clara Donnoli, Immacolata Dall’Oglio, Francesco Scerbo, Alessandro Stievano, Gennaro Rocco, Maurizio Zega, Marzia Lommi

**Affiliations:** 1 Department of Biomedicine and Prevention, University of Rome Tor Vergata, 00133 Rome, Italy; valentina.vanzi@students.uniroma2.eu (V.V.); francesco.scerbo@uniroma2.it (F.S.); marzia.lommi@uniroma2.eu (M.L.); 2 Center of Excellence for Nursing Scholarship (CECRI), Board of Nursing (OPI) of Rome, 00136 Rome, Italy; genna.rocco@gmail.com (G.R.); maurizio.zega@opi.roma.it (M.Z.); 3 JBI Italy Evidence-Based Practice and Health Research Centre, 00136 Rome, Italy; m.demaria@unilink.it (M.D.M.); d.ivziku@policlinicocampus.it (D.I.); claradonnoli@gmail.com (C.D.); alessandro.stievano@gmail.com (A.S.); 4 Department of Life Science, Health, and Health Professions, Link Campus University, 00165 Rome, Italy; 5 Department of Healthcare Professions, Azienda Ospedaliera San Giovanni Addolorata, 00184 Rome, Italy; 6 Department of Health Professions, Fondazione Policlinico Universitario Campus Bio-Medico, 00128 Rome, Italy; 7 Research Unit Science of Health Professions, Bambino Gesù Children’s Hospital, IRCCS, 00165 Rome, Italy; immacolata.dalloglio@opbg.net; 8 Department of Clinical and Experimental Medicine, University of Messina, 98125 Messina, Italy

**Keywords:** pediatric to adult care, transition readiness, assessment tools, adolescents, type 1 diabetes mellitus, COSMIN methodology

## Abstract

**Highlights:**

**What are the main findings?**

- Ten diabetes-specific instruments assessing transition readiness in adolescents were identified, but overall psychometric evidence remains limited.
- Among available tools, only the “On TRAck” instrument showed moderate-quality evidence with acceptable feasibility and reliability.

**What are the implications of the main findings?**

- Current transition-readiness assessment instruments should be used with caution in clinical practice due to incomplete validation and methodological limitations.
- Future research should prioritize longitudinal testing and predictive validity to improve assessment quality and ultimately support safer transition outcomes.

**Abstract:**

**Background**: Diabetes in youth, specifically type 1 diabetes (T1D), is an increasing global health concern. As prevalence rises, a growing number of adolescents are required to transition from pediatric to adult healthcare services. This phase is recognized as a particularly critical and high-risk period, during which emerging adults with T1D must exhibit advanced self-management skills to maintain optimal outcomes. When transition support is inadequate, the process is frequently associated with deterioration in glycemic control, higher rates of hospitalization, and significant psychological distress. **Methods**: A systematic review was conducted in accordance with PRISMA guidelines to identify and evaluate instruments that assess transition readiness in adolescents with diabetes, focusing on their psychometric properties. Five electronic databases (PubMed, CINAHL, Embase, APA PsycInfo, and Web of Science) were searched. Methodological quality and measurement properties were appraised using the updated 2024 COSMIN Guidelines. **Results**: Eleven studies were included, examining 10 distinct instruments. Overall, psychometric evidence was promising but limited. Only the “On TRAck” instrument demonstrated moderate-quality evidence with acceptable feasibility and reliability. Other tools showed partial support for validity, reliability, and responsiveness, but presented methodological limitations. **Conclusions**: Interest in diabetes-specific tools to assess transition readiness is growing, yet their psychometric robustness remains limited. Further research is needed to develop and validate instruments with stronger methodological rigor. Future efforts should focus on longitudinal performance and predictive validity to enhance their applicability in clinical practice and ultimately improve outcomes during transition.

## 1. Introduction

Diabetes is a global public health issue and youth-onset type 1 and type 2 diabetes are serious chronic conditions. Specifically, type 1 diabetes (T1D) is a chronic autoimmune condition representing one of the most common endocrine disorders in childhood and adolescence worldwide. Despite advances in treatment and monitoring technologies, people with T1D remain at risk for early complications, comorbidities, and excess mortality [1]. Diabetes’s negative impact on patients’ lives and systems is exacerbated by its prevalence, which has been rising steadily [2].

### 1.1. Global Epidemiological Trends of Type 1 Diabetes

The first global analysis of trends in diabetes prevalence and treatment coverage that covers all countries was recently published and presented a pooled analysis of 1108 population-representative studies with 141 million participants aged 18 years and older with measurements of fasting glucose and glycated hemoglobin (HbA1c), as well as information on diabetes treatment. Using a Bayesian hierarchical meta-regression model the study estimated 828 million (95% credible interval [CrI] 757–908) adults (those aged 18 years and older) with diabetes in 2022, with an increase of 630 million (554–713) from 1990 [3]. Similarly, several studies focusing on the epidemiology of diabetes in pediatric populations have been published. Zhang et al. conducted a cross-sectional study based on data derived from the Global Burden of Diseases (GBD) 2019, which included 204 countries and territories [4]. This analysis included a total of 1,449,897 children (aged 0 to 14 years) with diabetes. This study showed that cases of childhood diabetes increased by 39.37% (95% uncertainty interval [UI], 30.99–45.45%) from 1990 to 2019 [4]. Lawrence et al. conducted an observational, cross-sectional, multicenter study to evaluate trends in the prevalence of diabetes in children and adolescents in the United States (US) in the period 2001–2017. The authors considered a mean of 3.47 million youths for each prevalence year from 6 areas in the US. The estimated prevalence of T1D among those 19 years or younger increased significantly, from 1.48 per 1000 youths to 2.15 per 1000 youths. Referring to this analysis, the authors highlighted that the estimated prevalence of diabetes among children and adolescents increased significantly in recent years [1]. Although epidemiological studies often report data on diabetes overall, the increasing burden observed in pediatric populations is largely driven by type 1 diabetes. Consequently, the growing number of young individuals diagnosed with T1D highlights the need to better understand disease management and related health outcomes across the lifespan.

### 1.2. Transitional Care and Transition Readiness in Adolescents with Type 1 Diabetes

The process of transition from pediatric to adult health services represents a milestone in children and adolescents with T1D. During this period, there is a need for a shift in responsibility for a complex treatment regimen [5,6]. This process may be extremely challenging, because the management of this illness requires daily self-care, including monitoring blood glucose levels, adjusting and administering insulin, and making decisions about diet, physical activity and exercise. Moreover, emerging adults need to learn how to interact with the diabetes multidisciplinary team, to schedule appointments, and to communicate with insurance companies. Their parents previously managed all these activities, especially because most of the time, at diagnosis, initial education is often focused toward adult caregivers [7]. During this period of life, young adults experience different types of transition, changes in health care but also in lifestyle (e.g., education, occupation, living situation) and shifting relationships with family members, friends, and intimate others. All these conditions may be destabilizing for the individual [8]. The transition from pediatric to adult diabetes care represents a variable experience, but it is a high-risk period and the worsening of health outcomes during the transition process have been well documented. During this stage, around one in three young individuals with T1D reduce or cease their participation in diabetes management services [9]. There is a risk of both short- and long-term morbidity and mortality from ineffective transfer of care from pediatric to adult settings. Scientific literature underlined many negative outcomes related to the transition process: out-of-range glycemic control and high value of HbA1c, increased acute care utilization and hospitalizations, and increased stress and negative emotional outcomes on young adults and their families, are the most cited [8,10,11,12,13]. The American Diabetes Association (ADA) “Standards of Care in Diabetes” highlights how the management of diabetes in children and adolescents cannot simply be adapted from care routinely provided to adults with diabetes [14]. In this regard, transitional care is the term used to describe services that seek to bridge the care gap between children-focused and adult-centered services [15]. It defines “the purposeful, planned movement of adolescents and young adults with chronic physical and medical conditions from child-centered to adult-oriented health care systems” [16,17]. Given that, a lack of effective transition from pediatric to adult care may contribute to adverse outcomes in young adults with T1D. Assessing transition readiness is a crucial step in the transition process and suitable transition readiness measures are necessary to facilitate effective planning [18]. These instruments should be used to assess the level of readiness and to evaluate and direct the educational program in a measurable and comparable way. The importance of assessment tools for evaluating transition readiness in adolescents with chronic conditions is well established in the literature [15]. Although several systematic reviews are available on this topic, they lack a specific focus on diabetes [19,20,21]. Furthermore, research is advancing rapidly in this field, and several diabetes-specific studies have been published recently [22,23]. As a result, an update has become necessary to guide clinical practice and support healthcare professionals in facilitating the transition of adolescents and young adults with T1D, whose condition differs from other chronic diseases and whose management relies heavily on the individual’s adherence to daily self-care behaviors [24]. Moreover, in many chronic conditions, the effects of poor adherence may appear in the long term (i.e., hypertension, asthma) while, in diabetes, the consequences of management errors are immediate and potentially life-threatening (such as diabetic ketoacidosis or severe hypoglycemia). The overall purpose of this study was to provide a comprehensive overview of existing tools for assessing transition readiness in adolescents with T1D and to support clinicians and researchers in selecting psychometrically robust instruments for practice and research.

## 2. Materials and Methods

This systematic review aimed to assess the psychometric properties of self-reported instruments measuring transition readiness from pediatric to adults with diabetes, following the 2024 COSMIN Guidelines. The literature search was conducted across five databases, PubMed, CINAHL, Embase, APA PsycInfo, and Web of Knowledge, up to 13 February 2025. The search strategy was rerun in June 2025 prior to submission, and no additional eligible studies were identified. The search strategy adhered to the PRISMA-COSMIN statement [25,26] and incorporated filters recommended by Terwee et al. [27]. Key elements of the construct of interest (transition readiness, target population, and type of tool) were combined using Boolean operators (AND, OR and NOT). An example of the search strategy applied in PubMed is provided in Supplementary Materials Table S1.

Rayyan software (free version—essential tools, https://www.rayyan.ai/), a web platform that speeds up screening and organizing studies for systematic reviews, was used to facilitate the screening and selection process [28]. Studies were included if they focused on the development or validation of tools assessing transition readiness from pediatric to adults with diabetes. Studies in which the underlying condition of the population could be inferred were also considered; if more than 50% of the participants had diabetes, these studies were included. Only peer-reviewed articles published in English were considered. The literature search was not limited by publication time frame.

Exclusion criteria included studies that did not primarily evaluate the psychometric properties of instruments, such as cross-sectional research reporting only Cronbach’s α. Discussion papers and review protocols were also excluded due to their limited empirical contribution. Additionally, articles that did not provide the full instrument within the publication were omitted, in accordance with the 2024 COSMIN Guidelines, which require full access to the tool for proper evaluation. The systematic review protocol was registered in PROSPERO, an international registry where researchers publicly register systematic review protocols before starting the review to ensure transparency and avoid duplication.

To streamline the evaluation process, two researchers extracted data from the selected studies. The collected information included the instrument’s name, its authors, publication details (year and country), study type (validity or developmental), sample characteristics, number of items, response system, and measured psychometric properties. The review team utilized this data to outline the study characteristics and assess the psychometric properties of each instrument (Supplementary Materials Table S2).

The evaluation and synthesis process was structured into two phases: the first one, focused on assessing and summarizing evidence regarding the quality of development and validation studies, while the second phase examined and synthesized evidence on the measurement properties of the instruments. To facilitate data synthesis, the COSMIN checklists and an Excel^®^ file provided by the COSMIN research group were utilized. This Excel file has been reviewed by our research team to align with the 2024 COSMIN guidelines, as outlined in the new Manual Version 2.0, particularly with regard to the assessment of concept elicitation and cognitive interviews (Box 1) [29]. The original version was based on the 2018 COSMIN guideline criteria. In the first phase, four key steps were followed: (1) two independent reviewers assessed the methodological quality of each study using COSMIN Box 1 in the Excel^®^ file, evaluating the relevance of the instrument’s items, as well as the completeness and comprehensibility of the cognitive interview or pilot study conducted; (2) separately, two reviewers assessed the quality of the validation studies using COSMIN Box 2, examining the relevance, the comprehensiveness, and the comprehensibility of the patient-reported outcome measure (PROM) items from the perspective of both patients and experts; (3) the collected evidence was synthesized, and the instruments were evaluated to determine an overall score for relevance, comprehensiveness, comprehensibility, and content validity, rated from sufficient to inconsistent; (4) finally, the confidence in the quality of evidence and the reliability of the overall scores, classified as high, moderate, low, or very low, was determined using the modified Grading of Recommendations Assessment, Development, and Evaluation (GRADE) approach. The second phase followed four additional steps: (1) two independent reviewers evaluated the methodological quality of each study using COSMIN Box 3 in the Excel^®^ file; (2) separately, two reviewers assessed each measurement property based on the COSMIN checklist criteria, using COSMIN Box 4 in the Excel^®^ file; (3) the evidence for each instrument was synthesized with an evaluation of its psychometric properties (ranging from sufficient to indeterminate) and the quality of the evidence (high, moderate, low, or very low) following the GRADE approach; (4) finally, in accordance with the COSMIN 2024 guidelines [29], the instruments were classified into three categories based on the quality of the evidence: (1) recommended for use, if the PROM demonstrated high-quality evidence showing that all measurement properties were sufficient; (2) not recommended for use, if there was high-quality evidence that one or more measurement properties were insufficient; and (3) no recommendation could be made, for PROMs that did not meet the criteria for either of the previous two categories.

## 3. Results

### 3.1. Results of the Search Strategy

**Figure 1:**
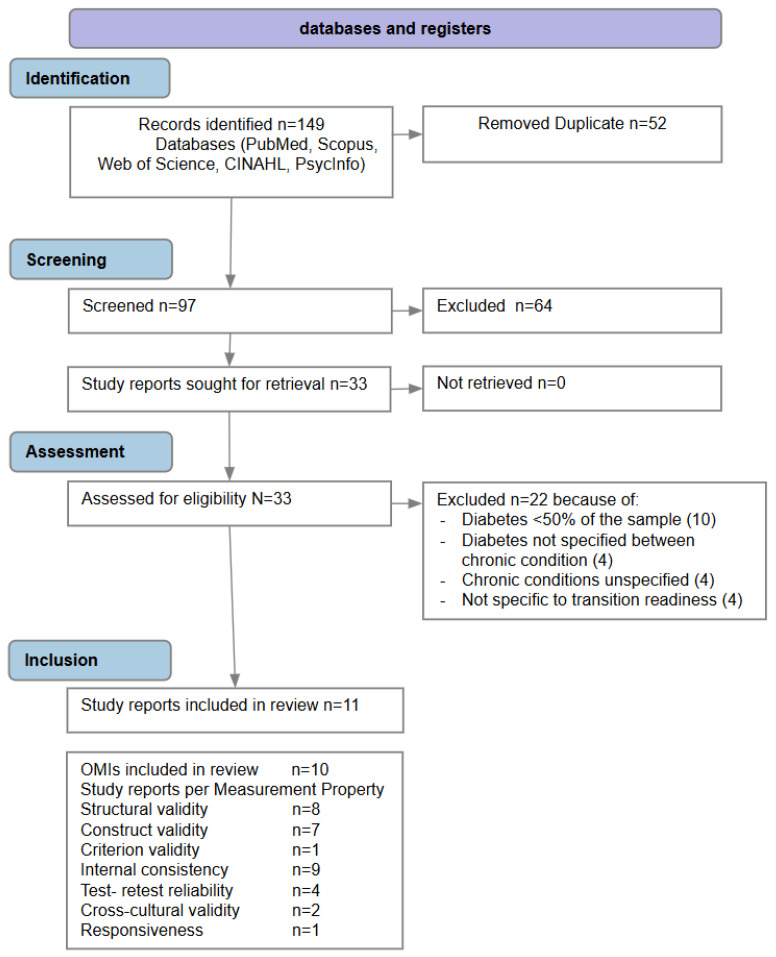
PRISMA Flow Chart of included studies [25].

A total of 149 records were identified through a systematic database search (PubMed, CINAHL, Scopus, APA PsycInfo, and Web of Science). After removing 52 duplicates, 97 records were screened based on title and abstract. Of these, 64 records were excluded as they did not meet the predefined inclusion criteria. Subsequently, 33 full-text articles were assessed for eligibility. Following full-text review, 22 articles were excluded for the following reasons: >50% of the sample did not have T1D or T2D (n = 10), diabetes was not specifically identified among the clinical conditions (n = 4), chronic conditions were not clearly specified (n = 4), the instrument was not specific to transition readiness (n = 4) (see Supplementary Materials Table S4). As a result, 11 articles were included in the qualitative synthesis, corresponding to the evaluation of 10 PROMs. See Figure 1.

### 3.2. Study Characteristics

**Figure 2:**
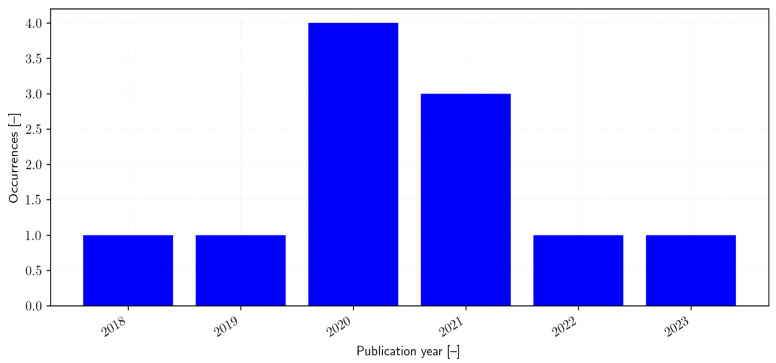
Temporal Distribution of Included Studies (Year of Publication).

**Figure 3:**
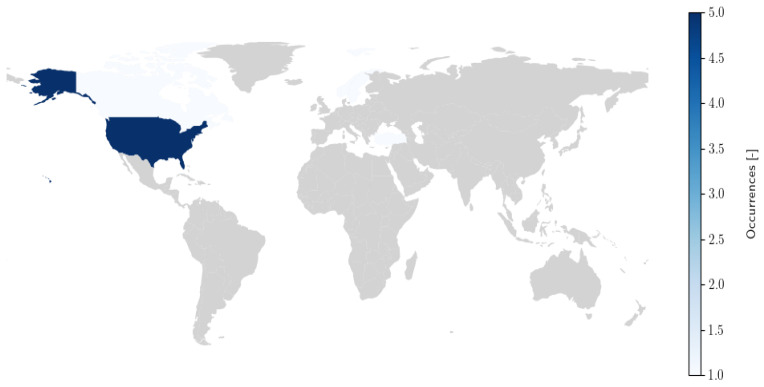
Geographic Distribution of the Included Studies (in the case of multicenter or multinational studies, each study was counted within all the geographical areas represented by the participating countries).

A total of 11 articles were included in the review, each addressing the development or validation of psychometric instruments designed to assess transition readiness in adolescents with diabetes. Of these, 5 studies focused on instrument development [30,31,32,33,34], while 2 were validation studies [35,36], and 4 combined both development and validation studies [37,38,39,40]. In total, 10 distinct instruments were identified across the included studies: the Diabetes Skills Checklist Teen-Report (DSC-T), Diabetes-Specific Risk-Taking Inventory (DSRI), Healthcare Transition Outcomes Inventory (HCTOI), the Good to Go (Good2Go), The On TRAck (On TRAck), Paediatric to Adult Diabetes Care Transition Questionnaire (PEDCaT-Q), the Readiness of Emerging Adults with Diabetes Diagnosed in Youth (READDY), Readiness for Independent Self-Care in Type 1 Diabetes-Teen version (RISQ-T), Transition Readiness and Experiences Questionnaire (TEXT-P) and the Transition Readiness Assessment Questionnaire (TRAQ). All tools focused on T1D. All these tools are featured in articles published in recent years, starting from 2018 to 2023 (Figure 2). From a geographical perspective, the majority of the studies were conducted in North America (n = 1 in Canada and n = 5 in USA (n = 2 in Florida, n = 1 in Washington, n = 1 in Texas, n = 1 in Illinois), followed by Europe (n = 1 in Sweden and n = 1 in Norway) and Asia (n = 1 in Turkey). Two studies involved collaborations between multiple countries and continents, such as from Canada and France [36] and Belgium and the UK [30]. These findings highlight a concentration of research activity in North America and Europe, with more limited representation from Asian contexts (Figure 3).

### 3.3. Study Quality and Content Validity

The quality of the included studies varied overall, with the majority showing initial attempts to establish content validity but lacking thorough methodological rigor. According to COSMIN criteria, only the HCTOI received sufficient scores with high evidence for comprehensiveness (+/H), and comprehensibility (+/H), based on expert panel input and cognitive debriefing with the target population. However, despite these strengths, the relevance and overall content validity was rated as inconsistent with high evidence (±/H). One critical limitation emerged in our appraisal: the Authors indicated for HCTOI a recall period of one year, which, according to COSMIN recommendations, may compromise the accuracy and reliability of patient-reported outcomes, particularly in adolescents and young adults, who may struggle to recall specific behaviors or experiences over such an extended timeframe. This introduces recall bias and compromises the instrument’s ability to accurately assess current transition-related competencies or needs. Referring to the DSC-T, despite its thoughtful development approach, the methodological quality of the validation study was rated as doubtful, primarily due to the limited documentation of cognitive testing procedures and the absence of explicit involvement of the target population (adolescents with T1D) in the concept elicitation phase. Moreover, there was no report of concept saturation or iterative item refinement based on patient feedback, which weakens the strength of the content validity evidence under COSMIN criteria. Most of the remaining instruments, including DSRI, Good2Go, On TRAck, READDY, TEXT-P, and TRAQ, showed consistent but limited evidence of content validity, with ratings sufficient but with low evidence across all domains. Although these tools included qualitative input from experts and adolescents, the procedures were often inadequate, such as having few participants in cognitive interviews, being poorly reported, or lacking formal concept elicitation from the target population. Notably, DSRI and On TRAck were developed with input from clinicians but relied on small adolescent samples (n = 4 and n = 3, respectively) for cognitive testing. TRAQ and TEXT-P demonstrated acceptable comprehensibility and cross-cultural adaptation but lacked a formal assessment of content saturation or patient-generated items. Overall, the findings emphasize the need for more rigorous and transparent development processes to enhance the content validity of transition readiness tools for adolescents with T1D. The RISQ-T, however, was not subjected to a formal concept elicitation phase involving adolescents—an essential step to ensure that the items reflect the lived experiences of the target population. No systematic procedures, such as conceptual saturation or alignment with established theoretical models, were conducted. Cognitive debriefing was reportedly carried out on a small and unspecified sample, with no details provided regarding the methods used, interview guides applied, or whether a structured analysis was performed. Notably, the PEDCaT-Q, despite being one of the few instruments developed and validated using a relatively large sample of emerging adults with T1D, received an overall content validity rating of +/L (sufficient, but supported by low-quality evidence). Although its development was informed by a literature review, focus group discussions, and cognitive interviews with 25 adolescents and young adults, the methodological process lacked sufficient transparency. Specifically, the number of experts involved in the content validation panel was not disclosed, documentation of concept saturation was absent, and the cognitive interview procedures were not described in adequate detail; for example, it remains unclear whether structured interview guides or probes were used (Supplementary Materials Tables S2, S3 and S5).

### 3.4. Tools, Psychometric Properties and Strength of Evidence

The psychometric evaluation of the included tools highlighted several methodological limitations that impacted the strength of evidence (Supplementary Materials Table S5).

The TRAQ is the most widely recognized tool in the literature, particularly in terms of the number of articles published about it and the linguistic validations it has been subjected to [41,42]. In the validation study conducted by Kızıler et al. (2018), the TRAQ was specifically tested on a population of adolescents and young adults with T1D, rather than on individuals with chronic conditions in general [35]. The tool consists of 20 items with a 5-point Likert response format across five domains. Internal consistency was high (α = 0.88), as was test–retest reliability (r = 0.79–0.93). Both principal component analysis (PCA) and confirmatory factor analysis (CFA) supported the underlying factor structure. However, the methodological quality of the study was rated as doubtful, although the psychometric performance of the tool appeared promising, and the strength of the evidence supporting its robustness was downgraded (Low).

Pierce and colleagues (2019, 2020) developed the HCTOI, following the rigorous standards embedded in NIH’s Patient Reported Outcomes Measurement Information System (PROMIS). It represents a multidimensional measure of outcomes of the transition from pediatric to adult healthcare for young adults affected by T1D. This instrument includes 34 items distributed across five domains [32,33]. Most items use a 5-point Likert scale, with a few employing dichotomous or numerical response formats. Structural validity, evaluated using CFA, did not show acceptable model fit for all domains. Despite moderate-to-good internal consistency (subscale α = 0.62–0.87), the primary limitation was the sample size (n = 128) in relation to the number of items—considered inadequate for assessing structural validity according to COSMIN standards. Therefore, although the instrument presents high-quality evidence, both its structural validity and internal consistency do not meet COSMIN criteria, and they are rated as insufficient. As a result, this is the only tool in the current evaluation for which a recommendation against its use has been issued.

The Good2Go tool, presented by Mellerio and colleagues in 2020, comprises 20 items distributed across three dimensions, using a 5-point Likert scale to assess transition readiness. The instrument showed acceptable internal consistency (α = 0.72–0.85) and test–retest reliability (ICCs between 0.70 and 0.80) [36]. However, the overall evidence was downgraded due to the low methodological quality of the study. Factor analysis (EFA and IRT/Rasch) was performed, but some items demonstrated misfit, weakening the structural validity.

The READDY was developed by Corathers and colleagues, in 2020 [38]. It represents an assessment tool with diabetes-specific content that provides patient-reported confidence in four fields: Diabetes Knowledge, Health System Navigation, Insulin Self-Management, and Health Behaviors. It includes 44 items scored on a 5-point Likert scale. Structural validity was the only psychometric property evaluated. Although the CFA yielded acceptable fit indices, it was performed on a sample size that did not meet the thresholds defined by COSMIN for adequacy—specifically, a minimum of seven participants per item is required for a “very good” rating, while five per item corresponds to a “doubtful” rating.

In 2020, Goethals and colleagues developed the RISQ-T, a 20-item self-report tool assessing adolescents’ readiness for independent diabetes self-management across three domains: Knowledge (dichotomous scale), Behavior, and Perceived Importance (5-point Likert scales) [30]. Tested in 178 adolescents, it showed acceptable internal consistency (α = 0.78) and construct validity. However, the lack of factor analysis limits evidence of structural validity, and the test–retest reliability over six months was assessed without verifying construct stability, reducing the strength of psychometric support.

The DSC-T developed by Papadakis et al. (2021) consists of 23 items rated on a 5-point Likert scale, designed to evaluate adolescents’ perceived autonomy in managing daily diabetes self-care tasks [31]. Psychometric evaluation of the DSC-T was conducted on a large sample of 1155 adolescents aged 12 to 18 years, and the instrument demonstrated excellent internal consistency (Cronbach’s α = 0.96). Construct validity was supported by positive correlations with diabetes-related strengths (r = 0.57; *p* < 0.001), negative correlations with HbA1c (r = −0.14; *p* < 0.001), and strong alignment with the parent-proxy version (DSC-PT, r = 0.87). Exploratory factor analysis (EFA) identified an initial two-factor solution, but a final unidimensional structure was retained, explaining 56.72% of the variance after removing two items related to social-context management. Despite the acceptable psychometric indices often coexist with insufficient or inconsistently reported development methodologies. A more rigorous, transparent, and participatory approach is needed to ensure that transition-readiness measures adequately reflect the competencies and perspectives of adolescents living with T1D.

The DSRI, developed by Wassermann and colleagues in 2021, is a self-report tool aimed at assessing risk-taking behaviors in adolescents with T1D that could compromise their health outcomes. These behaviors include actions such as skipping insulin doses, neglecting blood glucose monitoring, or engaging in risky activities without checking glucose levels. The DSRI was created by a multidisciplinary team, including psychologists, endocrinologists, and nurse practitioners, to specifically address diabetes-related risk-taking behaviors. The DSRI consists of 34 items rated on a 6-point Likert scale measuring frequency of diabetes-specific risk-taking behaviors. Although internal consistency was high (α = 0.92), the study involved only 30 participants, which is insufficient for the number of items tested. According to COSMIN recommendations, for a quantitative study, the optimal sample size should be greater than 100 subjects, while a sample of 30 subjects may lead to questionable results. This is advised for a robust psychometric evaluation. Consequently, the evidence supporting the tool’s reliability and construct validity was downgraded due to the questionable quality of the study design and the underpowered sample.

In 2021, Hodnekvam and colleagues developed and validated the PEDCaT-Q, a 98-item tool assessing patients’ experiences with diabetes care during transition [39]. Items are distributed across seven dimensions (pediatric/adult doctor, nurse, individualized care, and preparedness for transition) and rated on a 5-point Likert scale, later transformed to a 0–100 scale. The psychometric evaluation of PEDCaT-Q demonstrated sufficient measurement properties for structural validity and internal consistency. However, the instrument’s test–retest reliability presented a notable limitation. Although ICC values ranged from 0.64 to 0.85, the subscale Adult Individualized Care yielded an ICC of 0.64, which falls below the COSMIN cutoff of 0.70 and is thus considered insufficient. This weakens the instrument’s temporal stability and reduces confidence in its reproducibility over time. Accordingly, based on COSMIN criteria, the evidence supporting psychometric characteristics was rated as moderate. Overall, the psychometric profile of the PEDCaT-Q suggests potential for use in evaluating transition experiences in T1D populations, though further methodological refinement is warranted to strengthen its measurement precision.

The diabetes-specific “On TRAck” transition readiness scale was proposed by Al Khalifah and colleagues in 2022. It consists of 24 items with 3 subscales—Self-efficacy, Autonomy and Support and Maturity—on a 10-point Likert scale. Criterion validity showed inconsistent results, while internal consistency, construct validity, and structural validity were rated as sufficient, despite the very small sample size used for PCA, which does not fully meet COSMIN standards. Nevertheless, the overall quality of the evidence was considered moderate.

In 2023, Vallmark and colleagues designed the TEXT-P, composed of 13 items across three dimensions using a 5-point Likert scale, showed acceptable internal consistency (total α = 0.87; subscales α = 0.79–0.82) and responsiveness (no floor/ceiling effects). Factor analysis supported a three-factor solution, with the sample size reasonable. All instruments were classified as “No conclusion—more research on quality,” except for one (HCTOI), which could not be recommended for use in its current form and was therefore assigned a “recommendation against its use”.

## 4. Discussion

This review focused on the tools available in the literature that have been validated for adolescents and young adults with T1D. Even if in the search strategy the reviewers include both T1D and T2D, the results highlighted only studies on T1D. This gap is likely related to the different risk profiles of the two conditions: in adolescents with T1D, poor adherence and inadequate engagement during the transition process can lead to severe and immediate clinical consequences, whereas in T2D the complications, although serious, tend to manifest over a longer time horizon. According to the inclusion criteria, it is possible to highlight tools specifically designed for diabetes as well as general tools that have also been tested on people with T1D. Transition readiness can differ depending on the self-management needs and skill development required for a specific chronic condition, as well as individual characteristics [43]. These factors may not be fully reflected in a general assessment tool [38]. As outlined by several Authors, diabetes presents specific disease-features and a distinct risk compared to other chronic diseases, due to the immediate and life-threatening outcomes that can arise if the patient fails to properly manage insulin doses and adhere to the recommended diet. This awareness may lead to the need for developing specialized tools that focus on the specific skills and behaviors required for self-care in diabetes, rather than those applicable to other chronic conditions. Tools analyzed in this review are included in articles published very recently, in the past ten years, and this highlights an emerging need for assessment tools for transition readiness and a growing attention in research in this direction. In terms of how the tools are completed, they all involve questionnaires that the patient is asked to fill out. While self-assessment plays a key role in managing chronic conditions, some studies suggest that what patients report may not always align with the actual situation [44,45]. To address this issue, some researchers have created different versions of the same tool, often tailored for parents or caregivers, as well as for healthcare professionals who are involved in the patient’s care [46]. This approach, which involves multiple perspectives, can help ensure that the response to the assessment is more accurate and reliable [47,48].

Among the instruments evaluated for assessing transition readiness and experiences in adolescents and young adults with T1D, 3 diabetes-specific tools emerged as the most comprehensive: PEDCaT-Q, HCTOI, and On TRAck (Supplementary Materials Table S6). Their ranking was based on the breadth and depth of domain coverage, the number of subdomains assessed, and the quality of psychometric evidence reported according to COSMIN criteria. The PEDCaT-Q was identified as the most comprehensive instrument, specifically developed for young adults with T1D. It encompasses 7 dimensions and 24 subdomains, including therapeutic adherence, doctor–patient relationship, diabetes knowledge, family involvement, and continuity of care. Structural validity and Internal consistency were sufficient with moderate evidence, while reliability was insufficient for one dimension (adult individualized care). Furthermore, a key limitation of the PEDCaT-Q is its length, which may reduce its feasibility in clinical or large-scale settings. Nonetheless, based on COSMIN criteria, the tool was rated as having moderate-quality evidence and remains the most detailed and psychometrically supported instrument currently available. The HCTOI ranked second in terms of comprehensiveness. Also developed specifically for the T1D population, the HCTOI encompasses 17 subdomains across 7 domains, such as integration, self-management, navigation, and parental support. Its development process included expert consultation and cognitive interviews with young adults. While its conceptual structure is well-defined, psychometric limitations significantly weakened its overall quality. In particular, both structural validity and internal consistency were rated as insufficient under COSMIN, due to a small sample size for CFA and low reliability coefficients. The use of a one-year recall period further compromised its accuracy, potentially introducing recall bias. As a result, despite its broad theoretical coverage, the HCTOI was the only instrument to receive a formal “recommendation against its use” in its current form.

In third place, On TRAck stands out as a concise and diabetes-specific tool that assesses 15 subdomains across 7 key domains, including autonomy, support, and maturity. It includes 24 items and demonstrated strong psychometric properties: internal consistency was high (α = 0.88), subscales ranged from α = 0.73 to 0.82, and PCA confirmed a three-factor structure explaining 80% of the total variance. Construct validity was supported by associations with clinical indicators such as HbA1c and age. Although the validation sample was small (n = 115), which limited the strength of the evidence, the tool was rated as having moderate-quality evidence. Its brevity and ease of use make it particularly well-suited for clinical settings, where time and respondent burden must be considered.

In conclusion, all three instruments are diabetes-specific and address the unique challenges of transition in youth with T1D. The PEDCaT-Q offers the most extensive and detailed assessment, though at the cost of length and complexity. The HCTOI, while conceptually broad, is limited by insufficient psychometric support and is not currently recommended for use. On TRAck, although less exhaustive, provides a well-balanced and psychometrically sound alternative for both clinical and research applications.

### Limitations

This review presents some limitations that should be acknowledged. First, there was substantial heterogeneity in the methodological approaches and reporting quality of the included studies. While some instruments, such as PEDCaT-Q and On TRAck, described structured procedures for item development and validation, others lacked transparency regarding essential phases such as expert involvement, cognitive interviewing, or iterative item refinement. This variability limited the evaluation of content validity. Second, a single study reported data on responsiveness, a measurement property that is essential for determining whether a tool can detect meaningful clinical change over time. Moreover, no study was validated longitudinally to assess predictive validity or long-term outcomes. Another important limitation concerns the sample size used in the psychometric evaluation. Several studies relied on small or unbalanced samples, often composed of older adolescents. These limitations are especially critical for structural validation procedures such as CFA, where COSMIN recommends a minimum sample size equivalent to five to seven participants per item. The original search was completed in February 2025. Prior to submission, the search strategy was rerun in June 2025 to identify any newly published studies. No additional studies meeting the eligibility criteria were identified. Although this may reflect the time required to conduct validation studies in accordance with COSMIN standards, the increasing interest in this area suggests that further evidence may emerge in the near future. Therefore, periodic updates of the review will be warranted.

## 5. Conclusions

Throughout their lives, adolescents and young adults with T1D will inevitably need to transition from the pediatric setting to the adult-oriented healthcare system. The literature highlights how this transition period can pose a risk for these patients in terms of both acute and chronic complications. T1D has a unique risk profile considering the immediate and potentially fatal consequences that can occur if the patient does not adjust insulin administration and follow the appropriate diet. Assessing transition readiness has been recommended in numerous policy statements and guidelines. This scoping review identified ten diabetes-specific instruments designed to assess transition readiness in adolescents with T1D, but the overall psychometric evidence supporting these tools remains limited. Among the available measures, only the On TRAck instrument demonstrated moderate-quality evidence with acceptable feasibility and reliability. These findings indicate that current transition-readiness instruments should be used cautiously in clinical practice due to incomplete validation and methodological constraints. Nonetheless, readiness assessment alone cannot guarantee successful transition outcomes, which depend on a broader set of developmental and contextual factors. Future research should prioritize longitudinal designs and predictive validity testing to strengthen the evidence base and better understand the determinants of positive post-transfer health outcomes.

## Data Availability

No new data were created or analyzed in this study. The data supporting the findings of this study are available from the corresponding author upon reasonable request.

## Supplementary Materials

The following supporting information can be downloaded at: https://www.mdpi.com/article/10.3390/healthcare14050639/s1.

## Abbreviations

The following abbreviations are used in this manuscript:

T1D	Type 1 Diabetes
T2D	Type 2 Diabetes
DSC-T	Diabetes Skills Checklist Teen-Report
DSRI	Diabetes-Specific Risk-Taking Inventory
HCTOI	Healthcare Transition Outcomes Inventory
Good2Go	Good to Go
On TRAck	The On TRAck
PEDCaT-Q	Pediatric to Adult Diabetes Care Transition Questionnaire
READDY	Readiness of Emerging Adults with Diabetes Diagnosed in Youth
RISQ-T	Readiness for Independent Self-Care in Type 1 Diabetes-Teen version
TEXT-P	Transition Readiness and Experiences Questionnaire
TRAQ	Transition Readiness Assessment Questionnaire
