# Supplementary material for: A COSMIN Systematic Review of Transition Readiness Assessment Tools for Adolescents with Type 1 Diabetes
Source: Healthcare (Basel). 2026 Mar 3;14(5):639. doi: 10.3390/healthcare14050639 (PMC12984289; doi:10.3390/healthcare14050639)
Supplement: Supplementary file 1 [file healthcare-14-00639-s001.zip › healthcare-4150530-supplementary.pdf]

Supplementary Materials S1

Table S1. Example of search strategy on Medline (Pubmed)

**#1**	**Adolescent**<br><br><br><br><br>**Diabetes**	adolescent* OR “late adolescent*” OR “older adolescent*” OR “emerging adult*” OR “young adult*” OR “college student*” OR “high school student*” OR youth OR pediatric* OR paediatric* OR teenager* OR teen-ager* OR girl* OR boy OR minor* OR juvenile<br>“diabetes type 2” OR “diabetes mellitus type 2” OR “diabetes 2” OR t2dm OR “type two diabetes” OR “type two diabetes mellitus” OR dm2 OR “diabetes mellitus” OR “juvenile diabetes” OR “type 1 diabetes” OR “diabetes type 1” OR “diabetes mellitus type 1” OR “diabetes 1” OR “insulin dependent diabetic” OR t1dm OR “type one diabetes mellitus” OR “type 1 diabetes mellitus” OR “type 1 diabetes” OR “type one diabetes”	Title/Abstract
**#2**	**Readiness**	ready OR readiness OR preparation OR preparedness	All fields
**#3**	**Transition**	transition* OR “transition to adult care” OR “health care transition” OR “continuity of care” OR “transitional care”	All fields
**#4**	**Tools**	tool* OR scale* OR measure* OR instrument* OR questionnaire* OR survey OR checklist OR index OR assessment OR evaluation OR inventory OR score*	All fields
**#5**	**Filter**	(instrumentation[sh] OR methods[sh] OR “Validation Studies”[pt] OR “Comparative Study”[pt] OR “psychometrics”[MeSH] OR psychometr*[tiab] OR clinimetr*[tw] OR clinometr*[tw] OR “outcome assessment (health care)”[MeSH] OR “outcome assessment”[tiab] OR “outcome measure*”[tw] OR “observer variation”[MeSH] OR “observer variation”[tiab] OR “Health Status Indicators”[Mesh] OR “reproducibility of results”[MeSH] OR reproducib*[tiab] OR “discriminant analysis”[MeSH] OR reliab*[tiab] OR unreliab*[tiab] OR valid*[tiab] OR “coefficient of variation”[tiab] OR coefficient[tiab] OR homogeneity[tiab] OR homogeneous[tiab] OR “internal consistency”[tiab] OR (cronbach*[tiab] AND (alpha[tiab] OR alphas[tiab])) OR (item[tiab] AND (correlation*[tiab] OR selection*[tiab] OR reduction*[tiab])) OR agreement[tw] OR precision[tw] OR imprecision[tw] OR “precise values”[tw] OR test-retest[tiab] OR (test[tiab] AND retest[tiab]) OR (reliab*[tiab] AND (test[tiab] OR retest[tiab])) OR stability[tiab] OR interrater[tiab] OR inter-rater[tiab] OR intrarater[tiab] OR intra-rater[tiab] OR intertester[tiab] OR inter-tester[tiab] OR intratester[tiab] OR intra-tester[tiab] OR interobserver[tiab] OR inter-observer[tiab] OR intraobserver[tiab] OR intra-observer[tiab] OR intertechnician[tiab] OR inter-technician[tiab] OR intratechnician[tiab] OR intra-technician[tiab] OR interexaminer[tiab] OR inter-examiner[tiab] OR intraexaminer[tiab] OR intra-examiner[tiab] OR interassay[tiab] OR inter-assay[tiab] OR intraassay[tiab]	

		OR intra-assay[tiab] OR interindividual[tiab] OR inter-individual[tiab] OR intraindividual[tiab] OR intra-individual[tiab] OR interparticipant[tiab] OR inter-participant[tiab] OR intraparticipant[tiab] OR intra-participant[tiab] OR kappa[tiab] OR kappa's[tiab] OR kappas[tiab] OR repeatab*[tw] OR ((replicab*[tw] OR repeated[tw]) AND (measure[tw] OR measures[tw] OR findings[tw] OR result[tw] OR results[tw] OR test[tw] OR tests[tw])) OR generaliza*[tiab] OR generalisa*[tiab] OR concordance[tiab] OR (intraclass[tiab] AND correlation*[tiab]) OR discriminative[tiab] OR "known group"[tiab] OR "factor analysis"[tiab] OR "factor analyses"[tiab] OR "factor structure"[tiab] OR "factor structures"[tiab] OR dimension*[tiab] OR subscale*[tiab] OR (multitrait[tiab] AND scaling[tiab] AND (analysis[tiab] OR analyses[tiab])) OR "item discriminant"[tiab] OR "interscale correlation*"[tiab] OR error[tiab] OR errors[tiab] OR "individual variability"[tiab] OR "interval variability"[tiab] OR "rate variability"[tiab] OR (variability[tiab] AND (analysis[tiab] OR values[tiab])) OR (uncertainty[tiab] AND (measurement[tiab] OR measuring[tiab])) OR "standard error of measurement"[tiab] OR sensitiv*[tiab] OR responsive*[tiab] OR (limit[tiab] AND detection[tiab]) OR "minimal detectable concentration"[tiab] OR interpretab*[tiab] OR ((minimal[tiab] OR minimally[tiab] OR clinical[tiab] OR clinically[tiab]) AND (important[tiab] OR significant[tiab] OR detectable[tiab]) AND (change[tiab] OR difference[tiab])) OR (small*[tiab] AND (real[tiab] OR detectable[tiab]) AND (change[tiab] OR difference[tiab])) OR "meaningful change"[tiab] OR "ceiling effect"[tiab] OR "floor effect"[tiab] OR "Item response model"[tiab] OR IRT[tiab] OR Rasch[tiab] OR "Differential item functioning"[tiab] OR DIF[tiab] OR "computer adaptive testing"[tiab] OR "item bank"[tiab] OR "cross-cultural equivalence"[tiab])	
**#6**	**NOT**	("addresses"[Publication Type] OR "biography"[Publication Type] OR "case reports"[Publication Type] OR "comment"[Publication Type] OR "directory"[Publication Type] OR "editorial"[Publication Type] OR "festschrift"[Publication Type] OR "interview"[Publication Type] OR "lectures"[Publication Type] OR "legal cases"[Publication Type] OR "legislation"[Publication Type] OR "letter"[Publication Type] OR "news"[Publication Type] OR "newspaper article"[Publication Type] OR "patient education handout"[Publication Type] OR "popular works"[Publication Type] OR "congresses"[Publication Type] OR "consensus development conference"[Publication Type] OR "consensus development conference, nih"[Publication Type] OR "practice guideline"[Publication Type]) NOT ("animals"[MeSH Terms] NOT "humans"[MeSH Terms]	

Table S2. Studies included in review and psychometrics properties of the included tools

Tools<br>Author/Year Publication<br>Country<br>Type of Study	Sample	N. of Items<br>Subscale<br>Response System	Validity	Internal Consistency	Other Psychometric Properties	Quality of studies
**DSC-T**<br>Papadakis et al., 2021<br>Chicago<br>(North America)<br>Development study	1155 adolescent with T1D<br>(aged 12-18 years)	23 items<br>Unidimensionality scale<br>5-point Likert scale (1 = strongly disagree, 5 = strongly agree)<br>Higher scores indicate greater perceived independence in managing daily diabetes self-care tasks	**Concept elicitation:** review of literature, semi-structured interviews with pediatric diabetes health care professionals to assess perceptions of the skills and knowledge necessary for optimal daily diabetes management. Two members of the research team listened to audio recordings of the interviews to identify key themes. Results from the literature review and interviews were integrated to inform the development of 23 specific items.<br>**EFA**: oblimin rotation, 2-factor solution identified 14 items loading onto one factor representing a range of general diabetes management skills, and 2 items loading onto a second factor, representing diabetes management in a social setting (item deleted)<br>**EFA**: oblimin rotation, 1-factor solution who explained 56.72 of variance	**Total α** = 0.96		**Doubtful**

			**Construct validity**: 1) positive correlation with diabetes strengths (r = 0.57; p < 0.001), 2) negative correlation with HbA1c (r = –0.14; p < 0.001), 3) DSC-T and DSC-PT strongly correlated (r = 0.87)			
**DSRI**<br><br>Wasserman et al., 2021<br><br>Texas<br><br>(North America)<br><br>Development study	30 adolescents with T1D (aged 15–19 years)	34-item<br><br>Unidimensionality scale<br><br>6-point Likert scale (5 = “daily,” 4 = “weekly,” 3 = “monthly,” 2 = “every few months,” 1 = “yearly,” or 0 = “never”) or “not applicable (N/A).”<br><br>Higher score indicating more frequent diabetes-specific risk-taking behavior	**Items generation:** Focus group and interviews with 11 health care providers for (5 endocrinologists, 2 endocrinology nurse practitioners, 4 psychologists)<br><br>**Cognitive debriefings interview** with 4 adolescents with T1D for relevance and comprehensibility<br><br>**Content validity** was supported by risk perception ratings from adolescents with TD1 (n = 30, 3.0–4.8), their parents (n = 28, 3.3–4.8), and diabetes health care providers (n = 13, 2.5–4.9), identifying items as moderately to very risky<br><br>**Construct validity:** DMQ as higher diabetes-specific risk-taking was associated with poorer diabetes self-management (r = –0.56, P <0.01), greater general risk-taking (r = 0.41, P <0.05), and higher A1C levels over the past year (r = 0.57, P <0.01), while diabetes self-management and general risk-taking were unrelated (r = –0.22, P >0.05)	**Total α** = 0.92		Doubtful

**Good2Go**<br><br>Mellerio et al., 2020<br><br>Canada and France<br>(North America and Europe)<br><br>Validation study	321 (55% with T1D) adolescents (mean age 16.4 yy; SD 1.5)	20-item<br><br>3 domains: Health self-advocacy, Knowledge about chronic condition and Self-management skills<br><br>5-point Likert scale (from 1 "Low readiness" to 5 "High readiness")<br><br>Higher scores indicate increased<br>readiness	**Cognitive interviews:** in 5 adolescent (13-16 yy) for relevance and comprehensibility<br><br>**EFA** (Promax rotation): 3-factor solution (health self-advocacy, knowledge about chronic conditions, and self-management skills) explaining over 90% of the total variance<br><br>**IRT/Rasch** (rating scale model): acceptable infit/outfit (0.7–1.3) for all items except item 17 (underfit) and item 6 (overfit); unidimensionality supported (eigenvalue < 2), no significant local dependence.<br><br>**Construct validity:** 1) gender Vs self-management skills (girls better performance, p=0.08), 2) TD1 Vs knowledge about chronic condition (better performance)	**Total α:** 0.78<br><br>**Subscales α:** 0.72-0.85 for 3 dimension	Backward and forward translation<br><br>**Test–retest reliability** (interval time 15 days) ICC Health self-advocacy: 0.76, Knowledge about chronic condition 0.70 and<br>Self-management skills 0.80		Doubtful
**HCTOI**<br><br>Pierce et al., 2019*<br><br>Orlando<br>(North America)	8 adolescents with T1D (aged 19–24 years)	54-items<br><br>5 domains: Navigation, Self-management, Integration,<br>ownership, and Parental support)<br><br>49 items with 5-point Likert scale (from 0	**Panel expert**: 10 healthcare professionals (2 combined pediatric/adult endocrinologists, 2 pediatric endocrinologists, 1 adult endocrinologist, and 5 pediatric health psychologists) for relevance and comprehensibility<br><br>**Cognitive interviews**: in 8 young adults (19-24 old) for relevance, and comprehensiveness				Very good

Development study		“almost never/not at all true” to 4 “almost always/very true”)<br>and 5 items with numeric response or dichotomous<br>Higher score indicated better perceived success of the transition				
HCTOI<br>Pierce et al., 2020*<br>Orlando<br>(North America)<br>Development study	128 young adults with T1D (mean age 22.22 yy; SD 1.92)	34-items<br>5 domains (Integration, Ownership, Parental Support, Continuity of care, and Collaborative Relationship)<br>29-item with 5-point Likert scale (from 0 “almost never/not at all true” to 4 “almost always/very true”)<br>and 5 items with numeric response or dichotomous	**CFA:** 4-factor solution model: After the removal of some items, fit indices were good/improved for Parental Support (CFI = 0.99, TLI = 0.99, RMSEA = 0.03, SRMR = 0.02), Ownership (CFI = 0.97, TLI = 0.94, RMSEA = 0.08, SRMR = 0.04), and Integration (CFI = 0.96, TLI = 0.94, RMSEA = 0.07, SRMR = 0.05). The Navigation factor showed poor fit (CFI = 0.76, TLI = 0.71, RMSEA = 0.14, SRMR = 0.09), and was therefore split into two factors (Continuity of Care and Collaborative Relationship)<br>**First-order CFA 5-factor solution:**<br>Fit indices were acceptable for Continuity of Care (CFI = 0.95, TLI = 0.92, RMSEA = 0.06, SRMR = 0.06). Although item 7 had a loading < 0.30, model fit worsened when it was removed, so the item was retained.	**Subscales α:** 0.62–0.87 for 5 dimensions		Very good

		Higher score indicated better perceived success of the transition	Fit indices for Collaborative Relationship were excellent (CFI = 0.99, TLI = 0.96, RMSEA = 0.05, SRMR = 0.04).<br><br>**Construct validity:** 1) significant correlations with PedsQ, DDS and DSMP-SR, 2) significant correlation between the Integration (t(126) -4.43, p<0.001), Collaborative Relationship (t(126) -2.37, p<0.02), Ownership (t(126) - 3.61, p <0 .001), and Parental Support (t(126) - 2.61, p<0.01)			
**On TRAck**<br><br>Al Khalifah et al., 2022<br><br>Canada<br><br>(North America)<br><br>Development and validation study	115 adolescent with T1D (mean age 15.83; SD 1.6)	24-items<br><br>3 dimension: Self-efficacy, Autonomy and Support & maturity<br><br>10-point Likert scale (from 0 “Not at all” to 10 “Totally”)<br><br>Higher scores indicate increased<br><br>readiness	**Panel expert**: 12 multidisciplinary experts in diabetes (diabetologists, dietitians, diabetes educators) for relevance, comprehensiveness<br><br>**Face validity:** 3 adolescents with T1D for relevance and comprehensiveness<br><br>**PCA:** with orthogonal equamax rotation, 3 factor solution and 80% of the variance explained<br><br>**Criterion validity:** convergent validity with TRANSITION-Q (r=0..60, p<0.001)<br><br>**Construct validity:** 1) significantly associated with lower HbA1c, 2) “Self-efficacy” subscale was associated with age	**Total α:** 0.88<br><br>**Subscales α:** 0.73-0.82 for 3 dimensions		Doubtful

			but not A1C; "Support & maturity" subscale was associated with A1C but not age			
**PEDCaT-Q**<br><br>Hodnekvam et al., 2021<br><br>Norway<br>(Europe)<br><br>Development and validation study	321 patients with T1D (mean age 22.9 ± 1.2 years)	98 items of which 13 by clinical and socio-demographic characteristics<br><br>7 dimensions: Paediatric care doctor, Paediatric care nurse, Paediatric individualised care, Prepared for transition, Adult care doctor, Adult care nurse and Adult individualised care<br><br>5-point Likert (from 1 "Not at all" to 5 "To a very large extent")<br><br>Scales were transformed to scores ranging from 0 to 100, with a higher score representing a better experience in transition readiness	**Items generation:** literature review and focus group interviews (eight young people with T1D)<br><br>**Panel expert:** (number expert not indicated) multidisciplinary group of experts from paediatric and adult diabetes care for relevance and comprehensibility<br><br>**Cognitive interview:** performed with 11 persons, and after minor adjustments were made, with another 14 young people in the target group for relevance and comprehensibility<br><br>**PCA**: oblique rotation, loading factors ≥ 0.40 (Paediatric care from 0.45 to 0.87;<br>Adult care from 0.60 to 0.93; Prepared for transition from 0.53 to 0.90)<br><br>**Construct validity:** 1) Mean HbA1c inverse correlations with Contact with paediatric doctors ($r = −0.21$, $p < 0.01$), Individualised treatment in paediatric care ($r = −0.23$, $p < 0.01$), 2) Overall satisfaction with adequate positive correlations with contact with paediatric doctors, nurses, and individualised treatment (r = 0.49–0.65, p <	**Total α:** 0.70<br><br>**Subscales α:** 0.76- 0.85	**Test-retest reliability:** (5-9 weeks) ICC 0.64 to 0.85 (0.64 Adult individualised care)	Doubtful

			0.01), Contact with adult doctors, nurses, and individualised treatment ($r = 0.61–0.74$, $p < 0.01$)			
**READDY**<br><br>Corathers et al., 2020<br><br>Seattle<br><br>(North America)<br><br>Development and validation study	104 patients with T1D (15-24 years, mean<br><br>19.42, SD 6.25)	44 items<br><br>4 dimensions: Diabetes knowledge, Health system Navigation, Insulin Self-Management, Health Behaviors<br><br>5-point Likert (from 1 "Haven't thought about it" to 5 "Yes, I can do this")<br><br>Higher scores indicated greater confidence in transition readiness	**Items generation:** item pool was generated after a review of the literature. The tool was used at the time of a routine in-person medical or social work visit for patients 15 years of age to identify gaps or barriers in transition preparation and facilitate a discussion about needs.<br><br>**CFA**: 1) Diabetes Knowledge (6 items) x^2 12.68, P 0.18, CFI 0.98, RMSEA 0.06, SRMR 0.04; 2) Health System Navigation (12 items) x^2 82.94, P 0.01, CFI 0.92, RMSEA 0.08, SRMR 0.09; 3) Insulin Self-Management (4 items) x^2 51.49, P 0.47, CFI 0.99, RMSEA 0.01, SRMR 0.02; 4) Health Behaviors (10 items) x^2 70.43, P 0.01, CFI 0.89, RMSEA 0.10, SRMR 0.12.<br><br>**Panel experts:** 10 members (clinicians,<br><br>researchers, social workers, diabetes educators, psychologists) for relevance and comprehensibility<br><br>**Cognitive interview:** patient and parent representatives (number not specified) for relevance and comprehensibility			Doubtful

**RISQ-T**<br><br>Goethals et al., 2020<br><br>Belgium and Cambridge (Europe and North America)<br><br>Development study	178 adolescents with T1D (ages 13–17 years, mean age 14.9, SD 1.3 years)	20 item<br><br>3-dimensions: Knowledge, Behavior and adolescent's Perceived Importance<br><br>Knowledge domain dichotomous response Behavior and Perceived Importance domains 5 point Likert (from 0 "Never", to 4 "Always" and from 0 "Not important" to 4 "Very important")<br><br>Higher scores indicated greater readiness for independent diabetes self-care	**Items generation:** based on diabetes self-care, TRAQ, adolescent development literature and developed by multidisciplinary team<br><br>**Cognitive debriefing:** a small number of patients.<br><br>**Construct validity:** 1) self-efficacy, parent involvement, adherence and burden (r = 0.26–0.54, p < .0001); 2) age and adolescents scored higher than parents (p < .0001)	**Total α:** 0.78<br><br>**Subscales α:** 0.51-0.81	**Test-retest reliability** (6-month interval) ICC = 0.66	Doubtful
**TEXT-P**<br><br>Vallmark et al., 2023<br><br>Sweden (Europe)	163 participants with T1D (mean age 20.3, SD 0.9)	13 items<br><br>3 dimensions: Autonomy and Participation, Transition and Transfer Preparation and Healthcare-provider Communication	**Item generation:** Literature review, expert clinical and research input, and adaptation from Six Core Elements and Mind the Gap tools<br><br>**Cognitive interview**: 6 emerging adults (18–19 y) for relevance and comprehensiveness	**Total: α =** 0.866<br><br>**Subscales α:** 0.787–0.816	**Responsiveness:** no participant had the highest or lowest possible score, proving no floor or ceiling effect	Doubtful

Development and validation study		5-point Likert (Strongly Disagree – Strongly Agree)<br><br>Higher scores indicated better experience of transitional care	**Panel expert:** (7 nurses, 8 diabetologists) CVI for relevance<br><br>**EFA:** with polychoric matrix, principal axis method, promax rotation; 3-factor solution, 60% variance explained			
**TRAQ**<br><br>Kızıler et al., 2018<br><br>Turkey<br><br>(Asia)<br><br>Validation study	109 adolescents/young adults with T1D (range 14-21 years, average 15.28 and DS 1.44 years)	20-items<br><br>5-dimensions: Managing medications, Keeping appointment, Tracking health issues, Talking with providers, Managing daily activities<br><br>5-point Likert scale (from 1 “No, I don’t know how to do this” to 5 “Yes, I always do this when I need to”)<br><br>Higher scores indicate increased readiness	**Panel expert**: 4 expert opinions (2 nursing academicians, 1 biostatistician, and 1 pediatric endocrinologist) evaluated the scale for comprehensibility<br><br>**PCA:** with orthogonal varimax rotation, factor loads between 0.47 and 0.83, 5 factor solution and 74% of the variance explained<br><br>**CFA**: good model fit ($\chi2/df = 2.49$; GFI = 0.90; AGFI = 0.92; CFI = 0.93; RMSEA = 0.06; RMR = 0.22)	**Total α:** 0.88<br><br>**Subscales α:** 0.89-0.75 for 5 dimension	Backward and forward translation<br><br>Test–retest reliability (interval time 3 weeks) Pearson’s coefficient ranged 0.79 - 0.93 ($p<0.01$)	Doubtful

Note: *= same study or same sample; A1C= Hemoglobin A1c; AGFI=Adjusted Goodness of Fit Index; CFA= Confirmatory Factor Analysis; CFI= Comparative Fit Index; CVI=Content Validity Index; DDS = Diabetes Distress Scale; DMQ= Diabetes Management Questionnaire; DS= deviation standard; DSMP-SR= Diabetes Self-Management Profile, Self-Report; DSRI= Diabetes-Specific Risk-Taking Inventory; EFA= Exploratory Factor Analysis; GFI= Goodness of Fit Index; HCTOI= Health Care Transition Outcomes Inventory; ICC= Intraclass Correlation Coefficient; IRT-Rasch= Item Response Theory - Rasch Model; PCA= Principal Component Analysis; PedsQ= Pediatric Quality of Life Inventory; READDY= Readiness Assessment of Emerging Adults with Diabetes Diagnosed in Youth; RMSEA= Root Mean Square Error of Approximation; SRMR= Standardized Root Mean Square Residual; T1D= Type 1 Diabetes; TEXT-P= Transitional care EXPeriences Questionnaire; TLI= Tucker-Lewis Index; TRAQ= Transition readiness assessment questionnaire; $\chi2$/df= Chi-square divided by degrees of freedom.

Table S3. Methodological weaknesses in the evaluation of content validity among the studies included in the review

Tool	Methodological weaknesses	Specific examples	Impact on content validity
**HCTOI**	Long recall period	Recall period of one year may introduce recall bias	Compromises accuracy and reliability of outcomes
**DSC-T**	Limited documentation of cognitive testing procedures	Lack of explicit involvement of adolescents in concept elicitation, no report of concept saturation	Weakens content validity evidence
**DSRI**	Small sample for cognitive testing	Only 4 adolescents participated in cognitive testing	Limited evidence for content validity
**On TRAck**	Small sample and inadequate procedures for cognitive testing	Only 3 adolescents participated, no follow-up refinement	Inadequate evidence of content validity
**TRAQ**	Lack of concept saturation assessment	No formal assessment of content saturation or patient-generated items	Weakens content validity
**PEDCaT-Q**	Lack of transparency in cognitive interview procedures	Unspecified sample size, no details on methods used	Low-quality evidence for content validity
**RISQ-T**	No formal concept elicitation phase	No concept elicitation involving adolescents, no conceptual alignment	Instrument may not reflect lived experiences of the target population
**PEDCaT-Q**	Inadequate description of cognitive testing	No structured interview guides, unclear cognitive procedures	Weakens evidence for content validity

Supplementary Materials S2

Table S4: Articles exclusion by full-text with reasons

N	Reference	Reason for exclusion
1	Chapados, P., Aramideh, J., Lamore, K., Dumont, É., Lugasi, T., Clermont, M. J., Laberge, S., Scott, R., Laverdière, C., & Sultan, S. (2021). Getting ready for transition to adult care: Tool validation and multi-informant strategy using the Transition Readiness Assessment Questionnaire in pediatrics. Child: care, health and development, 47(5), 645–653. https://doi.org/10.1111/cch.12872	Diabetes <50% of the sample
2	Chapados P, Provencher S, Aramideh J, Dumont É, Lugasi T, Laverdière C, Sultan S, Desjardins L. Transition Readiness Assessment Questionnaire: Skill gaps and psychosocial predictors of transition readiness among adolescents and young adults with chronic medical conditions. Child Care Health Dev. 2024 Jan;50(1):e13156. doi: 10.1111/cch.13156. Epub 2023 Aug 3. PMID: 37535469.	Diabetes <50% of the sample
3	Cohen, S. E., Hooper, S. R., Javalkar, K., Haberman, C., Fenton, N., Lai, H., Mahan, J. D., Massengill, S., Kelly, M., Cantú, G., Medeiros, M., Phillips, A., Sawicki, G., Wood, D., Johnson, M., Benton, M. H., & Ferris, M. (2015). Self-Management and Transition Readiness Assessment: Concurrent, Predictive and Discriminant Validation of the STARx Questionnaire. *Journal of pediatric nursing*, *30*(5), 668–676. https://doi.org/10.1016/j.pedn.2015.05.006	Diabetes <50% of the sample
4	De Cunto, C. L., Eymann, A., Britos, M. L., González, F., Roizen, M., Rodríguez Celin, M. L., & Soriano Guppy, E. (2017). Cross-cultural adaptation of the Transition Readiness Assessment Questionnaire to Argentinian Spanish. Adaptación transcultural del cuestionario de transición a la atención médica del adulto al castellano argentino. *Archivos argentinos de pediatria*, *115*(2), 181–187. https://doi.org/10.5546/aap.2017.eng.181	Chronic conditions unspecified
5	Ferris, M. E., Harward, D. H., Bickford, K., Layton, J. B., Ferris, M. T., Hogan, S. L., Gipson, D. S., McCoy, L. P., & Hooper, S. R. (2012). A clinical tool to measure the components of health-care transition from pediatric care to adult care: the UNC TR(x)ANSITION scale. Renal failure, 34(6), 744–753. https://doi.org/10.3109/0886022X.2012.678171	Diabetes <50% of the sample
6	Ferris, M., Cohen, S., Haberman, C., Javalkar, K., Massengill, S., Mahan, J. D., Kim, S., Bickford, K., Cantu, G., Medeiros, M., Phillips, A., Ferris, M. T., & Hooper, S. R. (2015). Self-Management and Transition Readiness Assessment: Development, Reliability, and Factor Structure of the STARx Questionnaire. Journal of pediatric nursing, 30(5), 691–699. https://doi.org/10.1016/j.pedn.2015.05.009	Diabetes not specified between chronic condition

7	Funes D, F., León L, F., & Valenzuela C, R. (2020). Assessment of knowledge and autonomy for the transition from adolescent toward adult care. Evaluación de conocimientos y autonomía para la transición de adolescentes hacia la atención de adultos. Revista chilena de pediatria, 91(5), 722–731. https://doi.org/10.32641/rchped.vi91i5.1519	Chronic conditions unspecified
8	González, F., Roizen, M., Rodríguez Celin, M. L., De Cunto, C., Eymann, A., Mato, R., García Arrigoni, P., Staciuk, R., Ugo, F., & Fano, V. (2017). Validation of the Argentine Spanish version of Transition Readiness Assessment Questionnaire for adolescents with chronic conditions. Validación español-argentina del cuestionario de transición a la atención médica del adulto en adolescentes con enfermedades crónicas. *Archivos argentinos de pediatria*, *115*(1), 18–27. https://doi.org/10.5546/aap.2017.eng.18	Chronic conditions unspecified
9	Hilliard, M. E., Iturralde, E., Weissberg-Benchell, J., & Hood, K. K. (2017). The Diabetes Strengths and Resilience Measure for Adolescents With Type 1 Diabetes (DSTAR-Teen): Validation of a New, Brief Self-Report Measure. Journal of pediatric psychology, 42(9), 995–1005. https://doi.org/10.1093/jpepsy/jsx086	Not specific to transition readiness
10	Huang, Y., Wang, H., Diaz-Gonzalez de Ferris, M., & Qin, J. (2023). Translation and validation of the STARx questionnaire in transitioning Chinese adolescents and young adults with chronic health conditions. Journal of pediatric nursing, 71, 111–119. https://doi.org/10.1016/j.pedn.2022.11.003	Diabetes not specified between chronic condition
11	Johnson, K., McBee, M., Reiss, J., Livingood, W., & Wood, D. (2021). TRAQ Changes: Improving the Measurement of Transition Readiness by the Transition Readiness Assessment Questionnaire. Journal of pediatric nursing, 59, 188–195. https://doi.org/10.1016/j.pedn.2021.04.019	Diabetes not specified between chronic condition
12	Klassen, A. F., Grant, C., Barr, R., Brill, H., Kraus de Camargo, O., Ronen, G. M., Samaan, M. C., Mondal, T., Cano, S. J., Schlatman, A., Tsangaris, E., Athale, U., Wickert, N., & Gorter, J. W. (2015). Development and validation of a generic scale for use in transition programmes to measure self-management skills in adolescents with chronic health conditions: the TRANSITION-Q. *Child: care, health and development*, *41*(4), 547–558. https://doi.org/10.1111/cch.12207	Diabetes <50% of the sample
13	Ma, J., Yu, Q., Ding, W., Zhang, T., & Zhang, Y. (2021). Psychometric properties of the 'Self-Management and Transition to Adulthood with Rx = Treatment Questionnaire' in Chinese children and young people with chronic diseases. *International journal of nursing practice*, *27*(2), e12880. https://doi.org/10.1111/ijn.12880	Diabetes <50% of the sample
14	Moynihan, M., Saewyc, E., Whitehouse, S., Paone, M., & McPherson, G. (2015). Assessing readiness for transition from paediatric to adult health care: Revision and psychometric evaluation of the Am I ON TRAC for Adult Care questionnaire. *Journal of advanced nursing*, *71*(6), 1324–1335.	Diabetes <50% of the sample

	https://doi.org/10.1111/jan.12617	
15	Morisaki-Nakamura, M., Suzuki, S., Kobayashi, A., Kita, S., Sato, I., Iwasaki, M., Hirata, Y., Sato, A., Oka, A., & Kamibeppu, K. (2021). Development and validation of a Japanese version of the TRANSITION-Q. Pediatrics international : official journal of the Japan Pediatric Society, 63(3), 270–278. https://doi.org/10.1111/ped.14398	Diabetes <50% of the sample
16	Nazareth, M., Hart, L., Ferris, M., Rak, E., Hooper, S., & van Tilburg, M. A. L. (2018). A Parental Report of Youth Transition Readiness: The Parent STARx Questionnaire (STARx-P) and Re-evaluation of the STARx Child Report. *Journal of pediatric nursing*, *38*, 122–126. https://doi.org/10.1016/j.pedn.2017.08.033	Diabetes <50% of the sample
17	Saßmann, H., Kim-Dorner, S. J., Framme, J., Heidtmann, B., Kapellen, T., Kordonouri, O., Krosta, K. M. E., Pisarek, N., & Lange, K. (2023). Psychometric properties of the German teen and parent versions of the Problem Areas in Diabetes Scale (PAID). Psychological assessment, 35(7), e31–e42. https://doi.org/10.1037/pas0001243	Not specific to transition readiness
18	Shapiro, J. B., Vesco, A. T., Weil, L. E., Evans, M. A., Hood, K. K., & Weissberg-Benchell, J. (2018). Psychometric properties of the problem areas in diabetes: teen and parent of teen versions. Journal of Pediatric Psychology, 43(5), 561-571.	Not specific to transition readiness
19	Sato, Y., Ochiai, R., Ishizaki, Y., Nishida, T., Miura, K., Taki, A., Tani, Y., Naito, M., Takahashi, Y., Yaguchi-Saito, A., Hattori, M., & Nakayama, T. (2020). Validation of the Japanese Transition Readiness Assessment Questionnaire. Pediatrics international : official journal of the Japan Pediatric Society, 62(2), 221–228. https://doi.org/10.1111/ped.14086	Diabetes not specified between chronic condition
20	Weissberg-Benchell, J., & Antisdel-Lomaglio, J. (2011). Diabetes-specific emotional distress among adolescents: feasibility, reliability, and validity of the problem areas in diabetes-teen version. Pediatric diabetes, 12.	Not specific to transition readiness
21	Wood, D. L., Sawicki, G. S., Miller, M. D., Smotherman, C., Lukens-Bull, K., Livingood, W. C., Ferris, M., & Kraemer, D. F. (2014). The Transition Readiness Assessment Questionnaire (TRAQ): its factor structure, reliability, and validity. Academic pediatrics, 14(4), 415–422. https://doi.org/10.1016/j.acap.2014.03.008	Chronic conditions unspecified
22	Zhong, Y., Gilleskie, D. B., van Tilburg, M. A. L., Hooper, S. R., Rak, E., Javalkar, K., Nazareth, M., Pitts, B., Ndugga, M., Jain, N., Hart, L., Bhansali, S., Richards, J., Detwiler, R. K., True, K., de Pomposo, A. S. F., & Ferris, M. E. (2018). Longitudinal Self-Management and/or Transition Readiness per the TRxANSITION Index among Patients with Chronic Conditions in Pediatric or Adult Care Settings. The Journal of pediatrics, 203, 361–370.e1. https://doi.org/10.1016/j.jpeds.2018.06.052	Diabetes <50% of the sample

Table S5: Evaluation of content validity and psychometric properties and development of recommendations for the development of the instruments

Tool	Relevance	Comprehensiveness	Comprehensibility	Overall Content Validity	Structural Validity	Internal Consistency	Other Measurement	Recommendation
**DSC-T**	±/L	+/L	+/L	±/L	+/L	+/L	Construct validity +/L	No conclusion – more research on quality
**DSRI**	+/VL	+/VL	+/VL	+/VL	—---------	+/VL	Construct validity +/VL	No conclusion – more research on quality
**GOOD 2 GO**	+/L	+/L	+/L	+/L	+/L	+/L	Cross-cultural +/L<br>Reliability +/L<br>Construct validity +/L	No conclusion – more research on quality
**HCTOI**	±/H	+/H	+/H	±/H	-/H	-/H	Construct validity +/H	Conclude with recommendation against its use
**ON TRACK**	+/L	+/L	+/L	+/L	+/M	+/M	Criterion validity -/M<br>Construct validity +/M	No conclusion – more research on quality
**PEDCaT-Q**	+/L	+/L	+/L	+/L	+/M	+/M	Reliability -/M<br>Construct validity +/M	No conclusion – more research on quality

**READDY**	+/L	+/L	+/L	+/L	+/M	—----------	—--------------------------	No conclusion – more research on quality
**RISQ-T**	±/VL	+/VL	+/VL	±/VL	—----------	-/L	Reliability -/L<br>Construct validity +/L	No conclusion – more research on quality
**TEXT-P**	+/L	+/L	+/L	+/L	+/M	+/M	Responsiveness +/M	No conclusion – more research on quality
**TRAQ**	+/L	+/L	+/L	+/L	+/L	+/L	Cross-cultural ?/L<br>Reliability +/L	No conclusion – more research on quality

Note: + = sufficient; − = insufficient; ? = indeterminate; ± = inconsistent ; H= hight; M = moderate; L = low; VL = very low

Table S6: Mapping of assessment tools by conceptual domains and sub-domains

**DOMAINS**	**SUB-DOMAINS**	**SCALE**	N ITEM
Diabetes Autonomy and Self-Management	Assumption of personal responsibility	DSC-T	6
		GOOD2GO	1
		HCTOI	1
		ON TRACK	2
		PEDCaT-Q	1
		RISQ-T	5
		TEXT-P	2
		TRAQ	1
	Management during critical events	DSC-T	8
		GOOD2GO	1
		HCTOI	2
		ON TRACK	6
		PEDCaT-Q	6
		READDY	12
		RISQ-T	2
		TEXT-P	1
		TRAQ	1

	Management in different contexts	DSC-T	2
		HCTOI	2
		ON TRACK	1
		RISQ-T	3
		TRAQ	3
	Routine and therapeutic adherence	DSC-T	5
		HCTOI	3
		ON TRACK	2
		PEDCaT-Q	6
		READDY	3
		RISQ-T	6
		TRAQ	2
Risk behaviour and safety management	Management errors	DSRI	19
	Intentional risks	DSRI	6
		HCTOI	1
	Security in sensitive situations	DSC-T	2
		DSRI	9
		HCTOI	1
		READDY	1

Communication with the Health Team	Independence in medical examinations	GOOD2GO	1
		ON TRACK	1
		PEDCaT-Q	2
		TEXT-P	1
	Active participation	GOOD2GO	2
		HCTOI	3
		ON TRACK	1
		PEDCaT-Q	2
		READDY	2
		RISQ-T	2
		TRAQ	1
	Doctor-patient relationship	GOOD2GO	4
		HCTOI	1
		PEDCaT-Q	19
		TEXT-P	5
		TRAQ	2
Diabetes Knowledge and Health Awareness .	Understanding one's own state of health and ability to describe it	DSC-T	3
		GOOD2GO	3
		READDY	5
		PEDCaT-Q	3

	Clinical knowledge of diabetes	DSC-T	4
		GOOD2GO	1
		ON TRACK	1
		PEDCaT-Q	9
		READDY	5
		RISQ-T	4
	Therapeutic education	GOOD2GO	2
		DSC-T	7
		HCTOI	2
		ON TRACK	3
		PEDCaT-Q	2
		READDY	6
Navigating the health system	Access and organisation of care	GOOD2GO	2
		HCTOI	3
		ON TRACK	2
		PEDCaT-Q	8
		READDY	1
		TRAQ	3
	Knowledge of services and use of resources	GOOD2GO	1
		HCTOI	2

		READDY	5
		TRAQ	4
	Management of health documentation	GOOD2GO	1
		HCTOI	1
		PEDCaT-Q	8
		RISQ-T	1
		TRAQ	2
Family relations and support	Gradual detachment from parents	HCTOI	1
		ON TRACK	4
		PEDCaT-Q	4
	Perception of family support	ON TRACK	1
		PEDCaT-Q	2
	Relationship with parents	HCTOI	1
		PEDCaT-Q	2
	Active role of parents in emotional and financial support	HCTOI	3
		PEDCaT-Q	1
		TRAQ	1
Social support and participation in life	Balancing diabetes and daily life	HCTOI	1
		PEDCaT-Q	3
	Peer involvement	HTOI	1

		PEDCaT-Q	6
		READDY	3
		TEXT-P	1
	Managing diabetes in public	DSC-T	2
		PEDCaT-Q	1
		READDY	1
Transition to adulthood and continuity of care	Knowledge of legal aspects	TEXT-P	1
		PEDCaT-Q	4
	Post-transition experience	TEXT-P	2
	Transition planning	GOOD2GO	2
		PEDCaT-Q	6
		TEXT-P	4
